# Three new species of Fancy Case caterpillars from threatened forests of Hawaii (Lepidoptera, Cosmopterigidae, *Hyposmocoma*)

**DOI:** 10.3897/zookeys.170.1428

**Published:** 2012-02-22

**Authors:** Akito Y. Kawahara, Daniel Rubinoff

**Affiliations:** 1 McGuire Center for Lepidoptera and Biodiversity, Florida Museum of Natural History, University of Florida, Gainesville, FL 32611-2710 USA

**Keywords:** Case-bearing, endemism, Hawaii, conservation, Microlepidoptera, moth, new species

## Abstract

The endemic Hawaiian moth genus *Hyposmocoma* includes 348 described species and perhaps twice as many that remain undescribed. The genus is unusual within Lepidoptera in that its larvae create distinctive silk cases in which they perambulate while protected and camouflaged. An extraordinary diversity of case types exists, and to date more than ten different types have been identified, each corresponding roughly to a separate evolutionary lineage. In this study, we describe three new species of *Hyposmocoma*: *Hyposmocoma ipohapuu* **sp. n.** from Big Island, *Hyposmocoma makawao* **sp. n.** from Makawao Forest Reserve in Mauiand *Hyposmocoma tantala* **sp. n.** from Mt. Tantalus, Oahu, all of which produce tubular purse cases during their larval stage. We also describe the female of *Hyposmocoma inversella* Walsingham, which was previously undescribed, and re-describe two closely related species, *Hyposmocoma auropurpurea* Walsingham and *Hyposmocoma nebulifera* Walsingham, neither which have been formally described in recent years. We present for the first time, primer sequences for a 705 bp fragment of CAD, designed for *Hyposmocoma* and relatives. The molecular phylogeny based on mitochondrial and nuclear loci demonstrates that all are distinct species. The discovery of a new, endemic species from Mt. Tantalus, an area with many invasive species, suggests that even relatively degraded areas in Hawaii would be worthy of active conservation efforts.

## Introduction

*Hyposmocoma* Butler, 1881 includes 348 described species endemic to the Hawaiian Islands. The archipelago is one of the most threatened species “diversity hotspots” in the world, and large endemic radiations, including *Hyposmocoma*, are thought to have already lost many of their species (e.g. Carson et al. 1990; Freed et al. 1987; Jordan et al. 2003; Zimmerman 1978). The total number of *Hyposmocoma* species may be greater than the radiation of *Drosophila* (Kaneshiro 1997), as many species in this moth genusappear to be endemic to a single valley or volcano within an island (Rubinoff and Schmitz 2010). It is therefore imperative that taxonomic and biodiversity studies describe and catalog the remaining *Hyposmocoma*, as they likely provide a unique and important perspective on diversification on isolated island systems.

Unlike most cosmopterigids which are internal feeders of leaves, seeds and stems (Hodges 1998), *Hyposmocoma* feed externally. Species in the genus have extraordinary, in some cases globally unique, life-histories and behavior. Some caterpillars are carnivorous, feeding on tree snails (Rubinoff and Haines 2005) and others are amphibious living above and under water along streams (Rubinoff and Schmitz 2010; Schmitz and Rubinoff 2011b). Larvae in the genus create silk “cases” in which they perambulate while protected and camouflaged. An extraordinary diversity of larval case types exists, and to date, more than ten different types have been identified (e.g., Rubinoff and Schmitz 2010; Schmitz and Rubinoff 2008; 2009; 2011a; Zimmerman 1978). In this paper, we focus on the “tubular-purse” case type, a monophyletic group basal to most other lineages, such as the burrito and cone cases (Rubinoff and Schmitz 2010).

The total diversity of tubular-purse case *Hyposmocoma* remains largely unknown because historic sampling has focused primarily on the adult and there are very few records of larvae. From our examination of specimens in the Bishop Museum, Honolulu (BPBM), University of Hawaii Insect Museum (UHIM) and Smithsonian National Museum, Washington D.C. (USNM), there are seven species of tubular purse case *Hyposmocoma* that have been described prior to this study. These include, but may not be limited to the following species on the following islands: *Hyposmocoma auropurpurea* Walsingham, 1907 (Oahu), *Hyposmocoma ekemamao* Schmitz & Rubinoff, 2009 (Laysan), *Hyposmocoma fuscopurpurea* Walsingham, 1907 (Maui), *Hyposmocoma inversella* Walsingham, 1907 (Oahu), *Hyposmocoma mokumana* Schmitz & Rubinoff, 2009 (Necker), *Hyposmocoma nebulifera* Walsingham, 1907 (Oahu), and *Hyposmocoma rubescens* Walsingham, 1907 (Kauai).

Here we describe three new species of tubular purse case *Hyposmocoma*, *Hyposmocoma ipohapuu* Kawahara & Rubinoff, sp. n., *Hyposmocoma makawao* Kawahara & Rubinoff, sp. n.and *Hyposmocoma tantala* Kawahara & Rubinoff, sp. n. from the islands of Oahu, Maui and Hawai’i. While it is best to incorporate new species descriptions as part of a thorough revision, we have chosen to describe new species separately because of the sheer diversity of *Hyposmocoma* and the urgent conservation need of the genus. We present the first molecular phylogeny of purse-cased *Hyposmocoma* to shed light on the relationships between purse-cased species and assess its phylogenetic placement. We chose an integrative approach of combining traditional morphological data with molecular characters (Dayrat 2005; Rubinoff 2006; Will et al. 2005). This is part of an ongoing project to document the extraordinary diversity and ecology of *Hyposmocoma* across the Hawaiian Islands (e.g. Rubinoff 2008; Rubinoff and Haines 2005; Rubinoff and Schmitz 2010; Schmitz and Rubinoff 2008; 2009).

## Methods

### Specimen preparation, dissection, and description

Genitalia preparation techniques and morphological terminology follow previous work on *Hyposmocoma* (e.g. Rubinoff and Haines 2006; Schmitz and Rubinoff 2008, 2009). The abdomen of the dried moth was placed in a 20% KOH solution at room temperature overnight. When fats were not fully dissolved, the KOH solution and abdomen was heated on a hot plate. Genitalia were dissected and transferred to a solution of lactic acid with Orange G to stain hard cuticle. Membranes of the female genitalia were subsequently stained with Chlorazol Black, fixed in a sequence of 30%, 70%, and 95% ethyl alcohol, and mounted on slides with Euparal. Genitalia of two specimens, a female of *Hyposmocoma ipohapuu* and a male of *Hyposmocoma makawao* were prepared at the beginning of this study and were stained only with Orange G. Forewing and case lengths were measured with a scale in the ocular of a Leica MZ16 stereomicroscope. Adult moths were photographed with a Canon D50 digital camera mounted to the microscope; genitalia were photographed with an Olympus QColor3 Camera mounted on an Olympus SZX10 stereomicroscope. Types of previously described *Hyposmocoma* were examined from the collections of the Bernice P. Bishop Museum (BPBM) and the University of Hawaii Insect Museum (UHIM) on the University of Hawaii Manoa campus. Non-type specimens were examined from these museums and the US National Museum of Natural History (USNM). Types of the new species described here are deposited in the UHIM. All specimens in this study were reared from larvae, which were fed carrots and fish flakes and stored in Petri dishes until emergence.

### Molecular phylogenetic analysis

We included sequences of eight species of tubular purse-cased *Hyposmocoma*: *Hyposmocoma auropurpurea*; *Hyposmocoma ekemamao*; *Hyposmocoma inversella*; *Hyposmocoma mokumana*; *Hyposmocoma nebulifera*; and three new species: *Hyposmocoma ipohapuu*, *Hyposmocoma makawao* and *Hyposmocoma tantala*. We also included sequences for six outgroups: *Hyposmocoma alliterata* Walsingham, 1907; *Hyposmocoma carnivora* Schmitz & Rubinoff, 2011a; *Hyposmocoma eepawai* Schmitz & Rubinoff, 2011b; *Hyposmocoma laysanensis* Schmitz & Rubinoff, 2009; *Hyposmocoma nihoa* Schmitz & Rubinoff, 2009; and *Hyposmocoma nohomaalewa* Schmitz & Rubinoff, 2011a; all known to be distantly related to purse-cased *Hyposmocoma* (Rubinoff and Schmitz 2010). Taxa were sequenced for three genes, the mitochondrial *Cytochrome c Oxidase I* (COI; 762 bp), and two nuclear genes: the *Carbomoylphosphate Synthase* domain of CAD (705 bp), and *Elongation factor 1-alpha* (EF-1**α**; 771 bp), a concatenated sequence totaling 2,238 bp. We included data from both nuclear and mitochondrial genomes as they have different processes of selection, recombination and inheritance (Rubinoff 2006; Rubinoff and Holland 2005). All sequences included in this study are listed along with Genbank accession numbers in Table 1.

**Table 1.** Taxa sequenced for the present study along with UH log, extract, and GenBank accession numbers. An asterisk indicates a species that constructs a tubular case.

**Species**	**UH Log No.**	**Extract No.**	**CAD**	**EF-1α**	**COI**
*Hyposmocoma alliterata* Walsingham, 1907	DR08J7H	DN112	GU560396	GU560714	GU560555
*Hyposmocoma auropurpurea* Walsingham, 1907*	DR09A3I	DN1315	JQ182760	-	JQ231052
	DR09J1A	DN1219	JQ182756	JQ231030	JQ231047
	DR09J1A	DN1329	JQ182763	JQ231038	JQ231058
	DR09J1A	DN1331	-	JQ231040	JQ231060
	DR09K1A	DN1202	JQ182754	JQ231028	JQ231045
	DR09K1A	DN1330	-	JQ231039	JQ231059
	DR10C17B	DN1262	JQ182758	JQ231032	JQ231049
	DR10C17B	DN1328	JQ182762	JQ231037	JQ231057
*Hyposmocoma carnivora* Schmitz & Rubinoff, 2011a	DR08F5A	DN50	GU560342	GU560660	GU560501
*Hyposmocoma eepawai* Schmitz & Rubinoff, 2011b	DR09B7B	DN156	JQ182764	JQ231041	JQ231061
*Hyposmocoma ekemamao* Schmitz & Rubinoff, 2009*	DR07I2F	PS09	GU560311	GU560631	GU560472
	DR09H6A	DN1193	JQ182753	JQ231027	JQ231044
*Hyposmocoma inversella* Walsingham, 1907*	DR09A3I	DN1314A	JQ182759	-	JQ231050
	DR09A3I	DN1314B	-	-	JQ231051
*Hyposmocoma ipohapuu* Kawahara & Rubinoff, sp. n.*	DR08K8C	DN121	GU560405	GU560723	GU560564
	DR08K8C	DN1321	-	JQ231036	JQ231056
	DR08K8C	KY2	-	-	JQ231063
*Hyposmocoma laysanensis* Schmitz & Rubinoff, 2009	DR07I2D	DN31	GU560320	GU560640	GU560481
*Hyposmocoma makawao* Kawahara & Rubinoff, sp. n.*	DR08K12A	DN114	JQ182752	JQ231026	JQ231043
	DR08K12A	DN1320	-	JQ231035	JQ231055
*Hyposmocoma mokumana* Schmitz & Rubinoff, 2009*	DR04I1	CP96	GU560267	GU560601	GU560442
	DR04I1	CP97	GU560268	GU560602	GU560443
	DR04I1	CP98	GU560269	GU560603	GU560444
*Hyposmocoma nebulifera* Walsingham, 1907*	DR09J2B	DN1220	JQ182757	JQ231031	JQ231048
	DR09A3K	DN118	GU560402	GU560720	GU560561
	DR09A3K	DN161	JQ182765	JQ231042	JQ231062
*Hyposmocoma nihoa* Schmitz & Rubinoff, 2009	DR07G10	TT05	GU560312	GU560632	GU560473
*Hyposmocoma nohomaalewa* Schmitz & Rubinoff, 2011a	DR09B12D	DN1215	JQ182755	JQ231029	JQ231046
*Hyposmocoma tantala* Kawahara & Rubinoff, sp. n.*	DR10H2I	DN1317	JQ182761	JQ231033	JQ231053
	DR10H2I	DN1319	-	JQ231034	JQ231054

Genomic DNA was extracted from all specimens using the DNeasy^TM^ animal blood and tissue extraction kit following recommended protocols (Qiagen, Inc., Valencia, CA). The tissue was digested at 56° C for 24 hours, 200 ml of EB buffer was used to elute the DNA and extracts were stored at -20° C. COI was amplified in a single fragment: the forward primer Jerry (CAA CAT TTA TTT TGA TTT TTT GG) and reverse primer Pat-2 (TCC AAT GCA CTA ATC TGC CAT ATT A; Simon et al. 1994), and for EF-1**α**, a single fragment with the forward primer Coma (GGC CCA GGA AAT GGG CAA AGG) and reverse primer Toes (GGA GTC WCC AGC KAC GTA ACC; Rubinoff 2008), and for CAD the primers Cue (GGG ATT TAG CAA AAT TCA AAC GC) and Eh (CCT ATC GCT CAT ATC ATA ATC GG). The primers Cue and Eh were not published previously and thus described here. Amplifications were performed in a total volume of 25 μl. Thermal profiles for COI and CAD (94°C for 1 min; 51°C for 1 min; 72°C for 2 min), differed from EF-1**α** (94°C for 1 min; 53°C for 1 min; 72°C for 2 min). Each reaction started with 2 min of denaturation at 94°C, for 34 cycles, and followed by a final elongation for 12 min at 72°C. All PCR-products were visualized on 1% agarose gel and purified using QIAquick® spin columns (Qiagen, Inc., Valencia, CA) according to the manufacturer’s protocol. Sequencing was conducted with an Applied Biosystems 3130xl Genetic Analyzer at the Greenwood Molecular Biology Facility, Pacific Biosciences Research Center, the University of Hawaii at Manoa. For each sample, both sense and anti-sense strands of PCR products were sequenced.Multiple sequence alignments were conducted using MAFFT ver. 6.843 (Katoh 2010) and assembled in Geneious ver. 5.1 (Drummond et al. 2010). Genbank accession numbers are listed in Table 1.

Phylogenetic analyses were conducted with maximum likelihood (ML) as implemented in GARLI 1.0 (Zwickl 2006), and in a Bayesian framework with the software MrBayes ver. 3.1.2 (Ronquist and Huelsenbeck 2003). We used jModelTest (Posada 2008) to determine the best substitution model which was always the General-Time-Reversible (GTR) model (Lanave et al. 1984; Tavaré 1986), incorporating among-site rate heterogeneity modeled according to a gamma (G) distribution (Yang 1994) while allowing for a proportion of invariable sites (I) (Gu et al. 1995). One thousand ML and bootstrap tree searches were conducted as implemented in GARLI. To expedite tree searches, we used Grid computing (Cummings and Huskamp 2005) through The Lattice Project (Bazinet and Cummings 2009). GARLI analyses were conducted using the Lattice Grid portal (Bazinet and Cummings 2011). Bayesian analyses were conducted for 5 × 10^7^ generations with default settings and 3 × 10^7^ generations were treated as burn-in and removed. Chain convergence was assessed with MrBayes and AWTY Online (Wilgenbusch et al. 2004).

## Species descriptions

### Hyposmocoma auropurpurea

Walsingham, 1907

http://species-id.net/wiki/Hyposmocoma_auropurpurea

Figs 1 11 18

#### Diagnosis.

This species is unique among species of *Hyposmocoma* because it has metallic purple wings with a narrow, diagonal orange band near the wing apex.

#### Re-description.

**Figures 1–6.**
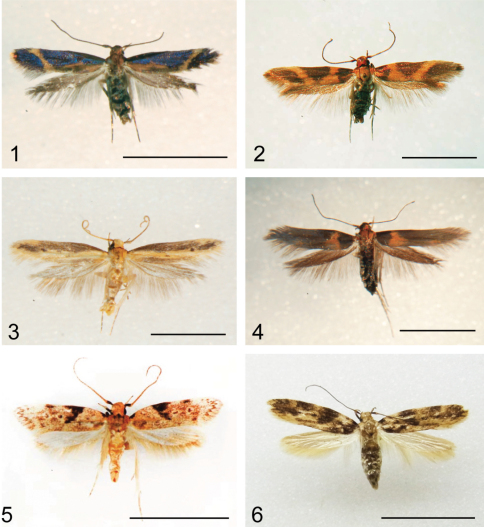
Purse-cased *Hyposmocoma*. **1** *Hyposmocoma auropurpurea*, male**2** *Hyposmocoma inversella*, male. **3** *Hyposmocoma ipohapuu* sp. n.holotype male **4** *Hyposmocoma makawao* sp. n.holotype male **5** *Hyposmocoma nebulifera*, male **6** *Hyposmocoma tantala* sp. n.holotypefemale. In order to examine genitalia, the abdomen of each was removed after photographs were taken. Scale bar = 5 mm.

 Male. (n = 2; Fig 1). Forewing length 4.8–5.0 mm.Head with a mixture of copper and metallic purple colored scales arranged radially from compound eye. Haustellum with light brown scales. Maxillary palpus reduced. Labial palpus curved with copper and metallic purple colored scales, scale color similar on all surfaces of palpus. Antenna brown with a mosaic of metallic purple scales. Thorax mostly copper; dark brown/purple scales present along anterior margin. Foreleg coxa with brown and metallic purple scales; femur, tibia, and tarsomeres mostly dark brown. Midleg as foreleg, but spurs covered in a mixture of dark and light-brown scales. Hindleg as midleg, but longer with long hairs on dorsal margin of tibia. Forewing metallic with a narrow diagonal orange band near wing apex. Diffuse orange patch in proximal region of FW along anal margin. Fringe orange and brown/metallic purple, longer scales tending to be brown/metallic purple, shorter ones orange. Hindwing brown with brown fringe. Abdomen dorsally dark brown; ventrally metallic brown/purple, with tuft of long dark brown scales covering lateral surface of genitalia.

**Male genitalia** (Fig. 11).Right brachium of uncus sickle shaped and curved at 1/2, tapering gradually, heavily sclerotized. Left brachium small, not sclerotized. Tegumen wide and sclerotized. Valvae roughly symmetrical, two long thin setae arising from approximately 2/3 length along dorsal margin of both valvae. Dense row of fine, hair-like setae disposed comblike along inner ventral margin of valva. Phallus stout, heavily sclerotized, open ventrally, tapered, and bent ventrad at about 1/2 of length; vesica without spines or cornuti. Anellus with two symmetrical rounded lobes with short setae.

**Female.** (n = 4). Externally as male, forewing length 5.2–5.5 mm.

**Female genitalia.** (Fig 18). Papillae anales very short and setose. Anterior and posterior apophyses slender, long, posterior apophyses about same length as anterior apophyses. Ostium bursae small, heavily sclerotized, externally protruding, roughly triangular, not snail-shaped. Corpus bursae oval and elongate, with very light scobination; signum absent. Ductus bursae long and narrow, slightly twisted, about equal in length of corpus bursae. Apical margin of tergum VIII with median emargination.

**Larval case** (n = 7).Dark brown, smooth, 7–9 mm in length and 1.7–2 mm wide.

#### Material examined.

 (2♂, 4♀). 1♂, 1♀: HI: Oahu, Waianae Range, Palikea trail, “purse” case 15-I-09, em. 23-III-2009, #DR09A3I, coll. P. Schmitz, D. Rubinoff, W. Haines, J. Eiben. 1♂, 1♀: HI: Oahu, Waianae Mountains, Palikea area, ~ elev. 850 on *Pittosporum* sp. leaves, 23-X-2009, em. 22-I-2010, #DR09J1A, coll. P. Krushelnycky. 1♀: HI: Oahu, Waianae Mountains, Palikea area, ~elev. 850 m, 5-XI-2009, em. 28-I-2010, #DR09K1A, coll. P. Krushelnycky. 1♀: HI: Oahu, South Waianae Mountains, Palikea area, 800–925 m, 30-III to 8-IV-2010, em 15-V-2010, #DR10C7B, coll. P. Krushelnychy and OANRP staff. All specimens from the UHIM.

#### Specimens sequenced.

Eight specimens from Oahu, Hawaii, USA (UH log numbers DR09A3I (1), DR09J1A (3), DR09K1A (2), DR10C17B (2)), extraction codes DN1202, DN1219, DN1262, DN1315, DN1328, DN1329, DN1330, and DN1331.

#### Biology.

Case-making larvae were collected from October to April. Larvae were typically found in leaf litter, but in some cases were found on leaves such as *Pittosporum* sp. (Pittosporaceae).

#### Distribution.

Known only from the Waianae Mountain Range, Oahu.

### Hyposmocoma inversella

Walsingham, 1907

http://species-id.net/wiki/Hyposmocoma_inversella

Figs 2 17

#### Diagnosis.

This species has a large, orange, “V”-shaped marking on the forewing found in no other described species of *Hyposmocoma*.

#### Re-description.

Male. (n = 1; Fig 2). Forewing length 4.9 mm.Head with copper-colored scales arranged radially from compound eye. Haustellum with light brown scales. Maxillary palpus reduced. Labial palpus curved with copper colored scales, scale color similar on all surfaces of palpus. Antenna brown with a mosaic of copper scales. Thorax mostly copper; dark brown scales present along anterior margin. Foreleg coxa with brown and copper scales; femur, tibia, and tarsomeres mostly dark brown with light brown ring at distal margin of femur, tibia, and tarsomeres I-V. Midleg as foreleg, but spurs covered in a mixture of dark and light-brown scales. Hindleg as midleg, but longer with long hairs on dorsal margin of tibia. Forewing brown, two transverse orange fascia form a “V”. Proximal orange band widens along anal margin, distal band uniform in width. Fringe orange and brown, longer scales tending to be brown, shorter ones orange. Hindwing brown with brown fringe. Abdomen dorsally dark brown; ventrally copper, with tuft of long dark brown scales covering lateral surface of genitalia.

**Male genitalia.** Right brachium of uncus sickle shaped, slender, heavily sclerotized, widening at 1/2 length, slightly twisted to left. Left brachium small, not sclerotized. Tegumen wide and sclerotized. Four long thin setae along dorsal margin of left valva, three shorter setae at same location of right valva. Left valva slightly wider than right. Dense row of fine, hair-like setae disposed along inner ventral margin of valva. Phallus stout, blunt tipped, heavily sclerotized, open ventrally, and bent ventrad at about 1/2 of length; vesica without spines or cornuti. Anellus with two symmetrical rounded lobes with short setae.

**Female.** (n = 1). Externally as male, forewing length 4.7 mm.

**Female genitalia.** (Fig 17). Papillae anales short and setose. Anterior and posterior apophyses slender, long, anterior apophyses slightly longer than posterior apophyses. Ostium bursae small, heavily sclerotized, externally protruding, roughly triangular, not snail-shaped. Corpus bursae oval and elongate, with very light scobination; signum absent. Ductus bursae long and narrow, slightly twisted, about equal in length of corpus bursae. Apical margin of tergum VIII with median emargination.

**Larval case.** (n = 2).Dark brown, smooth, 7.8–8.5 mm in length and 1.8–2 mm wide. The larval case is very similar to that of *Hyposmocoma auropurpurea*. Though both species are found in the Waianae Mountains, adult morphology and DNA sequence confirms these are distinct species.

#### Material examined.

 (1♂, 2♀). 1♂: HI: Oahu, Waianae Range, Palikea Trail, “purse” case, 15-I-2009, emergence. 16-III-2009, #DR09A3I, coll. P. Schmitz, D. Rubinoff, W. Haines, J. Eiben. Specimen in perfect condition. Male genitalia slide #AK100. 1♀: same data as male. Female genitalia slide #AK101. 1♀: HI: Oahu, Pahole NAR. Northern Waianae Mts. Elev. 500 m, on *Pipturus albidus* (Urticaceae). 14-XII-2010, #DR10L1C, em. 24-III-2011, coll. P. Krushelnycky. Additional voucher collection #AR0803, spec/lot #PKSP11751. Molecular ID: AK-259-11. All specimens from the UHIM.

#### Specimens sequenced.

Two specimens from Oahu, Hawaii, USA (UH log number DR09A3I), extraction codes DN1314A, DN1314B.

#### Biology.

Case-making larvae were collected in the Waianae Mountains of Oahu on the Palikea Trail during December and January. Larvae were found in leaf litter and on rotting logs. Adult emergence occurred between March and April. Because we have collected fairly extensively in the Waianae Mountains, we predict that this species univoltine, with larvae active during the winter months and adults emerging in the spring.

#### Distribution.

Known only from the Waianae Mountain Range, Oahu.

### Hyposmocoma ipohapuu

Kawahara & Rubinoff sp. n.

urn:lsid:zoobank.org:act:DBFC2894-9314-41EE-81BB-F19198CA3F6F

http://species-id.net/wiki/Hyposmocoma_ipohapuu

Figs 3 7 14 19

#### Diagnosis.

*Hyposmocoma ipohapuu* has a forewing pattern that differs from any other described species in the genus. A wide pale yellow band is present along the anal margin of the forewing and curves proximally at the wing margin towards the costa. A small, round, dark brown mark is present approximately two thirds of the way from the wing base to the apex.

#### Description.

**Male** (n = 2; Fig. 3). Forewing length 5.0 mm.Head with pale brown scales radiating from compound eye. Haustellum with a mixture of light and dark brown scales. Maxillary palpus reduced. Labial palpus recurved and covered in pale brown scales, which are dark laterally. Antennal flagellum pale brown, with a ring of dark brown scales extending from the proximal margin of each flagellomere. Thorax mainly pale brown, dark brown at cervical margin and near posterior margin of notum. Foreleg dark brown with pale brown ring at distal margin of femur. Midleg as foreleg, but with pale brown ring at distal margin of tibia and tarsomeres I-V, spurs pale brown. Hindleg as midleg but longer with long pale brown hairs. Forewing pale brown, with dark brown band extending from costal margin of wing base to apex, mark forming a convex arch near apex. A separate brown band extends along anal margin of forewing. Fringe pale brown. Hindwing and hindwing fringe grayish brown. Abdomen dorsally dark brown; ventrally pale brown with a tuft of long pale brown scales on either side of genitalia.

**Figures 7–10.**
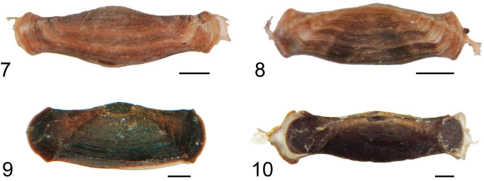
Larval cases of some purse-cased *Hyposmocoma*. **7** *Hyposmocoma ipohapuu* sp. n. **8** *Hyposmocoma makawao* sp. n. **9** *Hyposmocoma nebulifera* **10** *Hyposmocoma tantala* sp. n. Scale bar = 1 mm.

**Male genitalia.** (Fig. 14). Right brachium of uncus sickle shaped, rather thick, heavily sclerotized, slender at apical 1/3, slightly twisted to left. Left brachium small, not sclerotized. Tegumen wide and sclerotized. Valvae asymmetrical, left valva slightly wider at middle than right, with three long sclerotized club-shaped setae along dorsal margin curving posteriorly. Dorsal margin of right valva smooth, lacking setal sockets. Phallus stout, blunt tipped, heavily sclerotized, and bent ventrad at about 1/2 of length; vesica without spines or cornuti. Anellus with two symmetrical rounded lobes, thin until apex, both adorned with few small setae.

**Figures 11–15.**
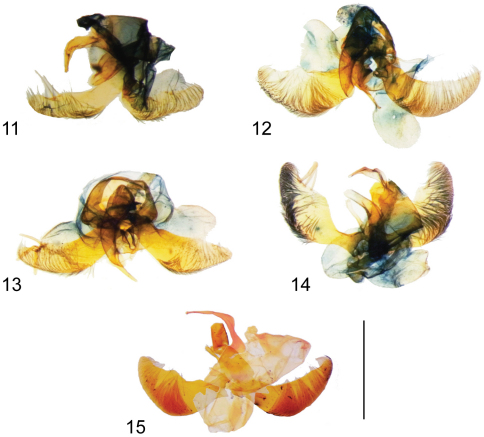
Male genitalia. **11** *Hyposmocoma auropurpurea* **12** *Hyposmocoma nebulifera* **13** *Hyposmocoma tantala* sp. n. **14** *Hyposmocoma ipohapuu* sp. n. **15** *Hyposmocoma makawao* sp. n. Scale bar = 1 mm. Figs **11–14** stained with Chlorazol Black, Fig. **15** stained with Orange G.

**Female.** (n = 1). Externally as male, but with longer forewing length (5.8 mm) and small dark brown mark medially below dark band on forewing.

**Female genitalia.** (Fig. 19). Papillae anales long and setose. Anterior and posterior apophyses thin and straight, slightly enlarged at posterior end, posterior apophyses about 2x length as anterior apophyses. Ostium bursae small, heavily sclerotized, externally protruding into a triangle, not snail-shaped. Corpus bursae oval with light scobination; signum absent. Ductus bursae thick, approximately 1/2 length of corpus bursae. Apical margin of tergum VIII with median emargination.

**Figures 16–19.**
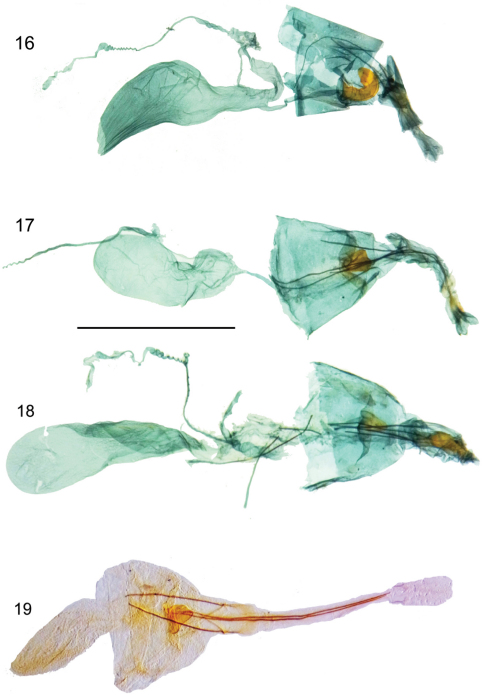
Female genitalia.**16** *Hyposmocoma tantala* sp. n., lateral view **17** *Hyposmocoma inversella*, ventral view **18** *Hyposmocoma auropurpurea*, ventral view **19** *Hyposmocoma ipohapuu* sp. n., ventral view. Scale bar = 1 mm. Figs **16–18** stained with Chlorazol Black, Fig. **19** stained with Orange G.

**Larval case.** (n = 5; Fig 7). The mature case is 5.9–6.6 mm in length and 1.2–1.5 mm wide, smooth with banding that follows the length of the case. The case widens slightly at both ends.

#### Material examined.

**Holotype:** ♂, [1] Printed white label: ‘H[AWAI]I: Hawaii, Volcano village | Pearl ave[nue], on tree fern | “purse” case, X-30-08 | em[ergence]. III-13-[20]08, #DR08K8C | coll[ector]. J[esse]. Eiben, M[elissa]. Dean’; [2] Printed red label: ‘HOLOTYPE | *Hyposmocoma ipohapuu* Kawahara & Rubinoff’. Male genitalia slide #AK110. Specimen in perfect condition. **Paratypes:** (1♂, 1♀). 1♂: same data as holotype but emergence 2-March-2009. 1♀: Data same as male paratype, but emergence 13-March-2009. Female genitalia slide #AK114. Cases (5): same data as holotype. All pinned specimens and slides deposited in the UHIM.

#### Specimens sequenced.

Three specimens from Big Island, Hawaii, USA (UH log number DR08K8C), extraction codes DN121, DN1321 and KY2. This species was tentatively called “Hsp69” before given this formal name.

#### Etymology.

This species is named “ipohapuu”, which means “tree-fern lover” in Hawaiian.

#### Biology.

Case-making larvae were collected on the abaxial surface of old fronds of a Hawaiian tree fern (Hāpu‘u, *Cibotium glaucum* (Sm.) Hook. & Arn., Cibotiaceae) in the rain forest at Volcano Village near Hawaii Volcano National Park, Hawaii (Big) Island. The habit of residing in old tree fern fronds, still attached to the stump, is typical of several purse case species including *Hyposmocoma filicivora* Meyrick, 1935.

#### Distribution.

Known only from Volcano Village, Hawaii Island. Probably restricted to the rainforest areas around Hawaii Volcanoes National Park, where it appears to be uncommon. The elevation for the type locality is approximately 1200 m.

### Hyposmocoma makawao

Kawahara & Rubinoff sp. n.

urn:lsid:zoobank.org:act:98EE02FA-5A90-4675-A4E2-A2236F8787FC

http://species-id.net/wiki/Hyposmocoma_makawao

Figs 4 8 15

#### Diagnosis.

*Hyposmocoma makawao* differs from any other species in the genus. No other species has a single, thick, transverse orange band near the base of the forewing.

#### Description.

**Male.** (n = 1; Fig. 4). Forewing length 4.8 mm. Head brown with iron-red scales near outer margin of eye; scales large near vertex. Haustellum pale brown. Maxillary palpus reduced. Labial palpus curved with pale brown scales, scales dark brown along lateral margin of labial palpus. Antennal flagellum dark brown along dorsal surface, lighter brown ventrally. Thorax reddish brown, laterally brown with patches of lighter brown scales below forewing. Foreleg and midleg with brown scales, scales dark brown laterally. Hindleg same as midleg, but with long scales along dorsal margin. Spines on legs light brown. Forewing dark brown with a single, wide fiery red-brown transverse fascia one fourth from the base of the wing to apex and narrowing towards costal margin. Abdomen covered in dark brown scales dorsally, light brown scales covering ventral surface.

**Male genitalia.** (Fig. 15). Right brachium of uncus sickle shaped, slender, heavily sclerotized, widening slightly at 1/2 length, slightly twisted to left. Left brachium small, not sclerotized. Tegumen wide and sclerotized. Valvae asymmetrical, left valva slightly wider than right, valvae without large sclerotized setae or sockets along dorsal margin. Dense row of fine, hair-like setae along inner ventral margin of both valvae. Phallus stout, blunt tipped, heavily sclerotized, open ventrally, and bent ventrad at about 1/2 of length; vesica without spines or cornuti. Anellus with two symmetrical rounded lobes with fine setae.

**Female.** (n = 1). Same as male, but with forewing length 4.9 mm.

**Larval case.** (n = 7; Fig. 8). The case is 4.1–5.0 mm in length and 1.2–1.6 mm wide, smooth with banding that follows the length of the case. Two wide, dark bands form a “V” that crosses over the central region of the case.

#### Material examined.

**Holotype:** ♂, [1] Printed white label: ‘H[AWAI]I: Maui, Makawao Forest Reserve | elev[ation]: 3500 ft, on Koa branches | “purse” case, I-30-[20]09, #DR08K12A | coll[ector]. W[illiam]. Haines’; [2] Printed red label: ‘HOLOTYPE | *Hyposmocoma makawao* Kawahara & Rubinoff’. Male genitalia slide AYK103. Specimen in perfect condition. **Paratype:** 1♀: same data as holotype, but emergence date 13-Feb-2009, abdomen missing. Cases (7): same data as holotype. All specimens stored in the UHIM.

#### Specimens sequenced.

Two specimens from Maui with UH log number DR08K12A, extraction codes DN114, DN1320. The specimen from which extract DN114 was taken was tentatively called “Hsp67” before being given this formal name.

#### Etymology.

This species is named “*makawao*” after its type locality, Makawao Forest Reserve, Maui.

#### Biology.

Case-making larvae were collected at Makawao Forest Reserve (MFR), Maui. Samples were collected using a beat-sheet, placed under branches of the koa tree (*Acacia koa* Gray).

#### Distribution.

Known only from the MFR, which harbors an extraordinary diverse natural fauna and flora, and is the only known locality for several species of *Hyposmocoma*, including *Hyposmocoma domicolens* (Butler, 1881), *Hyposmocoma molluscivora* Rubinoff & Haines, 2005, *Hyposmocoma opuulaau* Schmitz & Rubinoff, 2011, *Hyposmocoma pukoa* Schmitz & Rubinoff, 2011, and *Hyposmocoma pupumoehewa* Schmitz & Rubinoff, 2011. Unfortunately, the native habitat has been in sharp decline, even during the short course of this project, due to ongoing damage from invasive ungulates. If conservation action is not soon taken, it is likely that many of the rare plants and animals that remain in this mixed mesic forest will disappear.

### Hyposmocoma nebulifera

Walsingham, 1907

http://species-id.net/wiki/Hyposmocoma_nebulifera

Figs 5 9 12

#### Diagnosis.

*Hyposmocoma nebulifera* is similar to *Hyposmocoma rubescens* from Kauai, but differs in having a larger dark brown C-shaped forewing mark, and much smaller spurlike specialized setae on the valva. It is also similar to *Hyposmocoma ekemamao* but is larger, and has two central round spots on the forewing, while *Hyposmocoma ekemamao* only has one.

#### Re-description.

**Male.** (n = 3; Fig. 5). Forewing length 5.8 – 6.0 mm.Head light brown with scales near outer margin of eye, scales large near vertex. Haustellum pale brown. Maxillary palpus reduced. Labial palpus curved with pale brown scales, scales dark brown at terminus. Antennal flagellum light with dark brown bands. Thorax light brown, with lighter brown scales on tegula. Foreleg and midleg with brown scales and bands of light brown. Hindleg same as midleg, but with long scales along dorsal margin formed into a brush-like patch. Spines on legs light brown. Forewing light brown with a C-shaped dark brown mark 1/3 of distance to forewing apex, dark brown spot at base of forewing. One dark brown mark with light border at center of wing, another mark about 1/3 distance between first mark and apex. Abdomen covered in brown scales.

**Male genitalia.** (n = 1; Fig. 12). Right brachium of uncus sickle shaped, thin, long, and slightly twisted to left. Tegumen wide and sclerotized. Valvae asymmetrical, left valva medially slightly wider than right. Valvae without large sclerotized setae, but adorned with fine hair-like setae disposed comblike on inner surface of ventral margin. Phallus stout, blunt tipped, heavily sclerotized, and gradually curved ventrad at approximately 2/3 of length; vesica without spines or cornuti. Anellus with two symmetrical rounded lobes, thin until apex, both adorned with few small setae.

**Female.** (n = 2). Externally as male, but with larger wing span (6.5–7.2 mm).

**Female genitalia.** Papillae anales short and setose. Anterior and posterior apophyses thin, slightly curved, posterior apophyses slightly longer than anterior apophyses. Ostium bursae heavily sclerotized, externally protruding, C-shaped curled left, not triangular. Corpus bursae oval with light scobination; signum absent. Ductus bursae long, narrow, and approximately 2/3 length of corpus bursae. Apical margin of tergum VIII with median emargination.

**Larval case.** (n = 1; Fig. 9). Dark brown, smooth, 8.1 mm in length and 2.5 mm wide.

#### Material examined.

**Paratype:** ♀, [1] Printed white labels: ‘[HAWAII, Oahu,] Waianae Mts. | 3000 ft. OAHU | Hawaiian Is. | IV. 1892. | Perkins. 225133’; [2] ‘Fauna Hawaiiensis | Collection’; [3] Hand written and printed white label: ‘Hyposmocoma | nebulifera | Wlsm. | PARA-TYPE 1/3’; [4] BPBM Paratype pink label: ‘No 32412 | Hawaiian Coll. | BISHOP Museum’ (BPBM). **Non-type material:** 1♂, HI: Oahu, Waianae Mountains, Palikea trail, 15 January 2009, emergence 13 February 2009, #DR09A3K, coll. P. Schmitz, D. Rubinoff, W. Haines, J. Eiben, male genitalia slide #AK113 (UHIM). 1♂, 1♀: HI: Oahu, Waianae Mountains, Palikea area, elev: 850 m, in leaf litter, extracted in Berlese funnel, “purse case”, emergence 29 October 2009, #DR09J2B, coll. P. Krushelnycky, female genitalia slide #AK109 (UHIM).

#### Specimens sequenced.

Two specimens from Palikea, Oahu, UH log number DR09A3K, extraction codes DN118, DN161. One specimen from Oahu, UH log number DR09J2B, extraction code DN1220. The specimen from which extract DN118 was obtained was tentatively called “Hsp68”.

#### Distribution.

Known only from the Waianae Mountain Range, Oahu. With nearly thirty described species of *Hyposmocoma* (Zimmerman 1978), the Palikea preserve in the Waianae mountains has a very high diversity of *Hyposmocoma*, and the area is also one of the best-preserved on Oahu. The rich species diversity of *Hyposmocoma* may reflect the diversity throughout the island before human colonization.

### Hyposmocoma tantala

Kawahara & Rubinoff sp. n.

urn:lsid:zoobank.org:act:5B0155F3-0795-4706-895B-B728E4C575FC

http://species-id.net/wiki/Hyposmocoma_tantala

Figs 6 10 13 16

#### Diagnosis.

*Hyposmocoma tantala* is similar to *Hyposmocoma nebulifera*, but differs in having a dark forewing background color and much thicker dark forewing markings. The male genitalia has large sclerotized spines on the left valva that are absent in *Hyposmocoma nebulifera*.

#### Description.

**Male.** (n = 1). Forewing length 5.5 mm.Head light brown with light brown scales near outer margin of eye; scales large near vertex. Haustellum pale brown. Maxillary palpus reduced. Labial palpus curved with pale brown scales, scales dark brown at terminus. Antennal flagellum light with dark brown bands. Thorax light brown, with lighter brown scales on tegula. Foreleg and midleg with brown scales and bands of light brown. Hindleg same as midleg, but with long scales along dorsal margin formed into a brush-like patch. Spines on legs light brown. Forewing light brown with a large dark brown mark extending from costal margin 1/3 of distance to forewing apex and a smaller dark brown mark at 2/3 of distance to apex. Abdomen covered in brown scales.

**Male genitalia** (Fig. 13).Right brachium of uncus thick and curved ventrad, smooth, gradually tapering, slightly twisted to left. Left brachium small, not sclerotized. Tegumen wide and sclerotized. Shape of valva largely symmetrical, but left valva with three long tapered narrow spurlike setae along dorsal margin near apex; right valva without large sclerotized setae. Dense row of fine, hair-like setae disposed along inner ventral margin of both valvae. Phallus stout, blunt tipped, heavily sclerotized, open ventrally, and bent ventrad at about 1/2 of length; vesica without spines or cornuti. Anellus with two symmetrical rounded lobes with fine setae.

**Female.** (n = 2; Fig 6). Externally as male, but with larger wing span (6.2 mm).

**Female genitalia.** (Fig 16). Papillae anales short and setose. Anterior and posterior apophyses thin and straight, posterior apophyses slightly longer than anterior apophyses. Ostium bursae heavily sclerotized, externally protruding, and C-shaped curled left. Ductus bursae long and of small girth. Corpus bursae roughly kidney-shaped, with light scobination; signum absent. Apical margin of tergum VIII with median emargination.

**Larval case.** (n = 1; Fig 10). Dark brown, smooth, 9 mm in length and 2 mm wide.

#### Material examined.

**Holotype:** ♀, [1] Printed white label: ‘H[AWAI]I: Oahu, Tantalus, Manoa | Cliffs Trail, n[ea]r. Round Top Dr[ive]. | Purse Case DR10H2I [in bold type] | 22-Aug-2010, em[ergence]. 25-Oct[ober]-2010 | A. Y. Kawahara, W. Haines, | C. Yee, C. Atta collectors; [2] Printed red label: ‘HOLOTYPE | *Hyposmocoma tantala* Kawahara & Rubinoff’. Specimen in perfect condition. Female genitalia slide # slide AK108 (UHIM); **Non-type material:** 1♂, HI: Oahu: Mt. Tantalus, 4 km N of Manoa, 600 m., 8-VI-1991, coll. W. E. Steiner et al. (USNM).

#### Specimens sequenced.

Two specimens from Oahu (UH log number DR10H2I), extraction codes DN1317, DN1319.

#### Etymology.

This species is named “*tantala*” after Mt. Tantalus, from where the type specimen was collected. While Mt. Tantalus has experienced a tremendous amount of destruction from invasive species in the past century, it has historically been a locality with very high endemism, and the type locality of several other endemic insects, including flies (Hardy 1960; 1964), beetles (e.g. Liebherr and Zimmerman 2000), and at least two other species of *Hyposmocoma* (Zimmerman 1978). We wish to recognize this historically noteworthy site and draw attention to the fact that some endemic species, worthy of conservation, still persist even after long periods of disturbance.

#### Biology.

Adults were reared from case-making larvae collected on bark of a damp dead tree covered partially with lichen.

#### Distribution.

Known only from Mt. Tantalus, Oahu.

## Conclusion

Our morphological investigation coupled with molecular sequence data supports the separation of all three species described as new in this study. Interspecific genetic divergence in COI for sister-species for other Lepidoptera range from slightly less than 1% to nearly 4% (e.g. Landry et al. 1999; Rubinoff and Powell 2004; Sperling and Hickey 1994, but see also Mitter et al. 2011), suggesting that species of *Hyposmocoma* studied here exhibit relatively high levels of interspecific divergence. This may be due to lineage-specific rates of evolution, and/or differential rates of colonization and extinction in the Hawaiian islands. Future research will focus on the evolutionary processes behind the distribution and diversity of purse-cased *Hyposmocoma* and their relatives.

While we have sampled *Hyposmocoma* broadly on all Hawaiian islands, all species in this study and those previous appear to be largely restricted to one volcano on one island. Unfortunately, restricted ranges pose challenges for conservation since many species are vulnerable to extinction because of widespread habitat destruction. Mount Tantalus on Oahu, the only known locality for *Hyposmocoma tantala*, is the type locality for endemic flies, beetles and at least two other species of *Hyposmocoma*. The area is heavily infested with invasive species, but does retain some endemism, though the native habitat is still declining. The discovery of a new, endemic species from Mt. Tantalus suggests that even relatively degraded areas would be worthy of active conservation efforts to control invasive species. The same is true for the Makawao Forest Reserve on Maui that straddles an area between rainforest and dry forest, and thus supports a particularly diverse, highly unique endemic flora and fauna. This forest reserve is also the type locality for six species of *Hyposmocoma*. As such, native species in these refugia continue to decline, and this must include endemic species of *Hyposmocoma*, all of which are found nowhere else. We hope this information will encourage more active conservation, not only to protect these moths, but also the immense diversity of geographically restricted species displaying amazingly specialized life histories and the remnant native habitats that sustain them.

**Figure 20.**
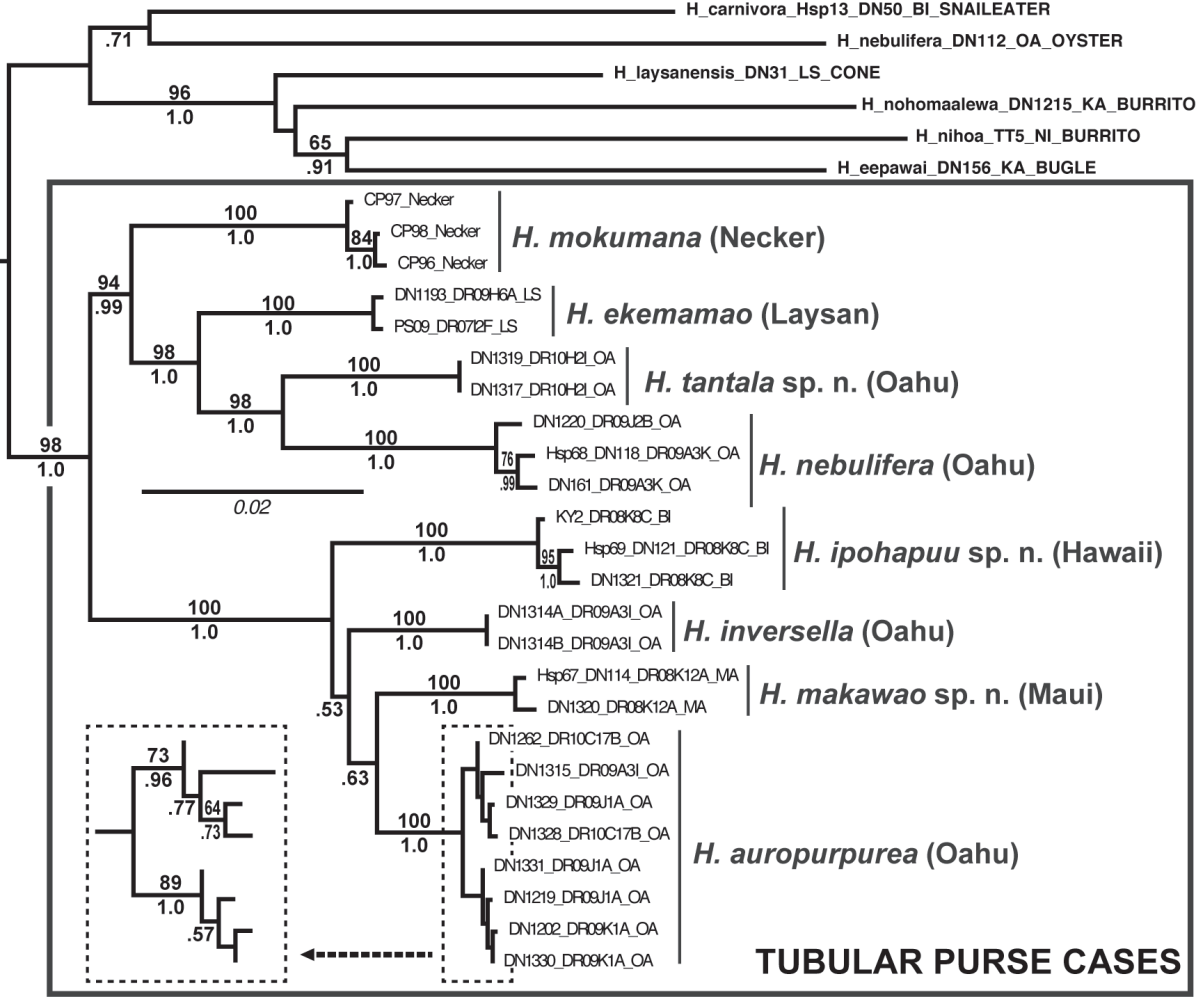
ML phylogeny of tubular purse-cased *Hyposmocoma* and relatives. Numbers above branches are ML bootstrap values, numbers below are Bayesian posterior probabilities. Scale bar = 0.02 substitutions/site.

## Supplementary Material

XML Treatment for Hyposmocoma auropurpurea

XML Treatment for Hyposmocoma inversella

XML Treatment for Hyposmocoma ipohapuu

XML Treatment for Hyposmocoma makawao

XML Treatment for Hyposmocoma nebulifera

XML Treatment for Hyposmocoma tantala
